# Chocolate for breakfast prevents circadian desynchrony in experimental models of jet-lag and shift-work

**DOI:** 10.1038/s41598-020-63227-w

**Published:** 2020-04-10

**Authors:** Carolina Escobar, Estefania Espitia-Bautista, Mara A. Guzmán-Ruiz, Natalí N. Guerrero- Vargas, Miguel Ángel Hernández-Navarrete, Manuel Ángeles-Castellanos, Brenda Morales-Pérez, Ruud M. Buijs

**Affiliations:** 1 Departamento de Anatomía, Facultad de Medicina, UNAM, Mexico City, Mexico; 2 Departamento de Fisiología, Facultad de Medicina, UNAM, Mexico City, Mexico; 3 Instituto de Investigaciones Biomédicas, UNAM, Mexico City, Mexico

**Keywords:** Neuroscience, Circadian rhythms and sleep, Circadian regulation

## Abstract

Night-workers, transcontinental travelers and individuals that regularly shift their sleep timing, suffer from circadian desynchrony and are at risk to develop metabolic disease, cancer, and mood disorders, among others. Experimental and clinical studies provide evidence that food intake restricted to the normal activity phase is a potent synchronizer for the circadian system and can prevent the detrimental metabolic effects associated with circadian disruption. As an alternative, we hypothesized that a timed piece of chocolate scheduled to the onset of the activity phase may be sufficient stimulus to synchronize circadian rhythms under conditions of shift-work or jet-lag. In Wistar rats, a daily piece of chocolate coupled to the onset of the active phase (breakfast) accelerated re-entrainment in a jet-lag model by setting the activity of the suprachiasmatic nucleus (SCN) to the new cycle. Furthermore, in a rat model of shift-work, a piece of chocolate for breakfast prevented circadian desynchrony, by increasing the amplitude of the day-night c-Fos activation in the SCN. Contrasting, chocolate for dinner prevented re-entrainment in the jet-lag condition and favored circadian desynchrony in the shift-work models. Moreover, chocolate for breakfast resulted in low body weight gain while chocolate for dinner boosted up body weight. Present data evidence the relevance of the timing of a highly caloric and palatable meal for circadian synchrony and metabolic function.

## Introduction

Modern human society is exposed to a 7/24 activity schedule, leading individuals to low sleep quality and disrupted daily sleep-activity rhythms. It is known from shift-workers and frequent travelers ailing from jet-lag, that disturbed sleep-wake schedules create a conflict between the circadian system and the temporal signals derived from the cyclic environment, such as the light-dark cycle^1^. This is further supported by clinical and experimental findings indicating that a conflict between external time signals and the internal temporal order transmitted by the suprachiasmatic nucleus (SCN) can lead to internal desynchrony^2^.

Circadian organization is necessary to prepare the organisms for the daily challenges and requires a well-coordinated synchrony with the day-night cycle. Epidemiological and experimental studies indicate that constant exposure to situations that induce circadian disruption, increase the risk to develop overweight, metabolic diseases, cardiovascular problems and cancer^3,4^.

Diverse strategies are used to prevent circadian disruption, including scheduled melatonin administration, scheduled dexamethasone administration, exercise or scheduled feeding^5–8^. Scheduled feeding has shown to be a strong entraining signal for the body; when food intake is synchronized with the activity phase it exerts beneficial effects on the circadian system and metabolism^8–10^. In experimental studies, timed food access restricted to the active phase accelerates resynchronization in a jet-lag model, prevents circadian desynchrony in a shift-work model^10^ and induces healthy effects in metabolism^11,12^. Conversely, food scheduled to the sleep/rest phase, exerts a disruptive influence on circadian synchrony, altering metabolism and behavior^11,13^.

We have previously reported that scheduled access to chocolate, entrains brain areas involved in motivation and in the metabolic response to food^14^. Scheduled chocolate also entrains the circadian system, enhancing the amplitude of neuronal activation in the SCN^15^. In the present study we hypothesized that a daily piece of chocolate scheduled in synchrony with the onset of the normal activity phase (breakfast) would be a powerful stimulus to prevent circadian disruption in experimental models of jet-lag and shift-work. Therefore, experimental models of jet-lag and shift-work were used to test the synchronizing effects of a piece of chocolate for breakfast or dinner on general activity, body temperature, daily c-Fos activation and metabolic indicators.

## Results

### Chocolate for breakfast accelerates re-entrainment in an acute jet-lag condition

Jet-lag results from a sudden change of the light-dark cycle due to transmeridional traveling, which leads to a misalignment between internal circadian rhythms and the external day-night cycle. The transitory days necessary for re-entrainment are associated with behavioral and physiological discomfort.

**Figure 1:**
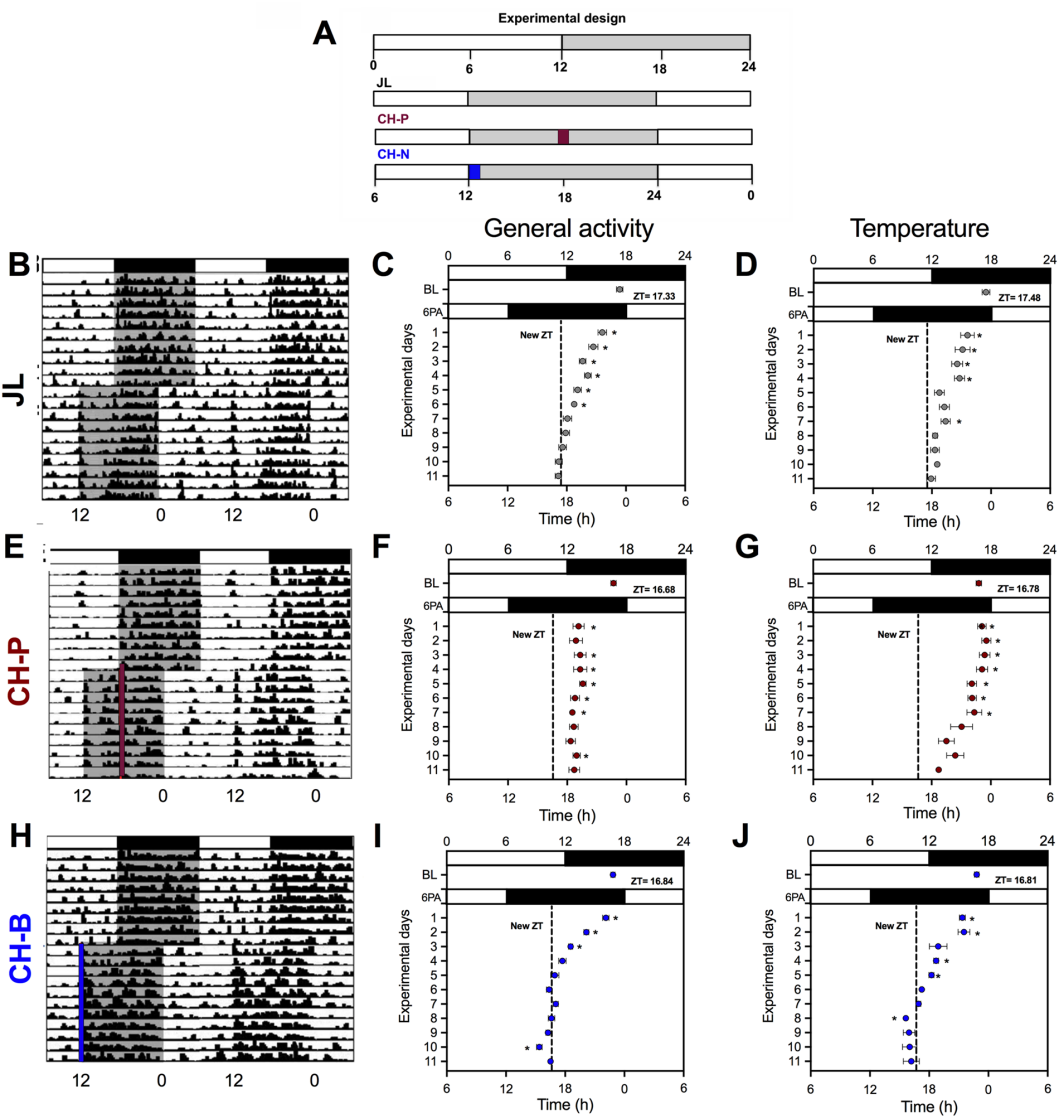
In a jet-lag condition a scheduled piece of chocolate for breakfast accelerates re-entrainment to a new light-dark cycle. (**A**) Experimental design for jet-lag, due to a sudden 6 h phase advance (6PA) without receiving chocolate (JL), or combined with chocolate access to the onset of the previous night (CH-P) or to the new night (CH-N). Days of re-entrainment for the JL group, (**B**) as seen with a representative actogram, (**C**) the map of acrophases for general activity and (**D**) the map of acrophases for body temperature. Days of re-entrainment for the CH-P group, (**E**) as seen with a representative actogram, (**F**) the map of acrophases for general activity and (**G**) the map of acrophases for body temperature. Chocolate time is indicated with a red line in the actogram. Days of re-entrainment for the CH-N group, H) as seen with a representative actogram, (**I**) the map of acrophases for general activity and (**J**) the map of acrophases for body temperature. Chocolate time is indicated with a blue line in the actogram. The dotted vertical line in the maps of acrophases indicates the new projected acrophase after the 6PA. Horizontal white and black bars over the X-axis and over the actograms indicate the 12 h day and 12 h night cycle. Data are expressed as the mean ± SEM (N = 8/group). The difference with the expected new acrophase was estimated with a one-way ANOVA for repeated measures followed by a Dunnett´s post hoc test P < 0.05.

To determine whether a piece of chocolate can influence re-entrainment in jet-lag conditions, adult Wistar rats were exposed to a sudden 6 h phase advance (6PA) of their light-dark cycle and received simultaneously 5 grams of chocolate either at the beginning of the new night (CH-N; representing breakfast) or at the beginning of the previous night (CH-P; wrong phase breakfast); they were compared with rats undergoing the jet-lag without receiving chocolate (JL; Fig. 1A). Successful re-entrainment was defined as reaching the new acrophase, thus reaching similar values to the 6 h projected acrophase and maintaining this phase relation of at least 3 consecutive days.

After a sudden 6PA the JL rats required 7 days to reach the new acrophase for general activity and 9 days for core temperature (Fig. 1B–D). Rats with access to chocolate scheduled to the previous night (CH-P) were unable to reach the expected acrophases for activity and temperature (Fig. 1E–G), while rats with access to chocolate in the new night (CH-N) reduced to 4 days the amount of days needed to reach the new acrophase in general activity and reduced to 6 days for core temperature (Fig. 1H–J). The one way ANOVA indicated a significant difference between groups in the number of days required for re-entrainment for general activity (F(2,21) = 59.34; P < 0.0001) and for core temperature (F(2,15) = 38.67: P < 0.001). The posthoc test indicated significant difference between all groups (P < 0.01).

Interestingly, the 5 grams piece of chocolate was ingested within 15 min, and this brief event had similar synchronizing effects as observed in animals exposed to a 12 h scheduled food access coinciding with the new activity phase (Supplementary Fig. 1E–G). Importantly, as observed in the CH-P rats, 12 h of food access scheduled to the previous night prevented re-synchronization (Supplementary Fig. 1B–D).

### A palatable meal accelerates re-entrainment

In order to identify which element of chocolate may have favored re-entrainment, another series of rats exposed to the jet-lag protocol were exposed for five days to scheduled access to one of the individual elements of chocolate. On the sixth day animals were left in constant darkness (DD) and did not receive the corresponding element of chocolate in order to observe the endogenous phase achieved with each component in general activity and core temperature.

**Figure 2:**
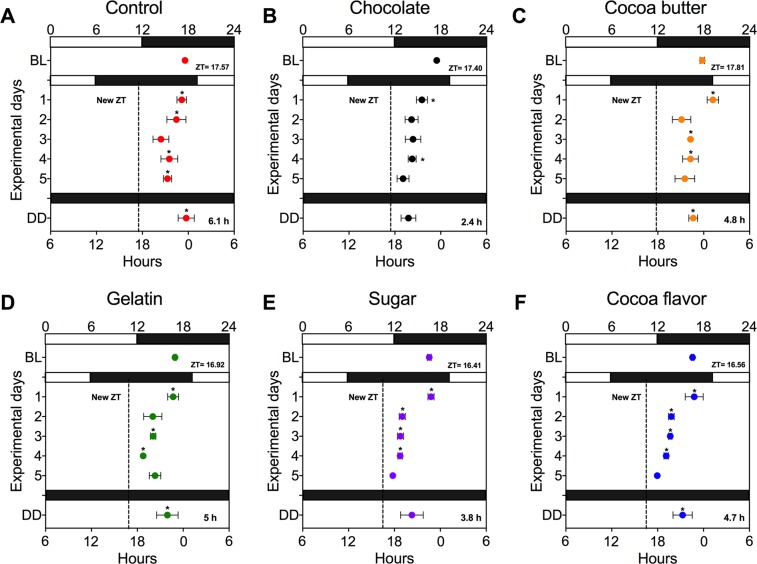
Entrainment of general activity by the different components of chocolate after a 6 h phase advance. (**A**) Rats were exposed to a sudden 6 h phase advance (6PA) by switching off the lights 6 hours before expected (control) and the acrophases for the following 5 days were estimated. On day 6 the lights were kept off in order to observe the circadian phase. (**B**) Simultaneous to the new night, rats received 5 g of chocolate, or pellets containing separate elements of a chocolate bar, (**C**) cocoa butter, (**D**) gelatin (which was used as the base to prepare all the pellets), (**E**) sugar and (**F**) cocoa flavor with splenda. Daily acrophases were compared with the expected new acrophase (vertical dotted line) and the difference between their current acrophase in DD and the new acrophase is indicated in the right-low corner of each graph. Horizontal white bars over the X-axis indicate lights on and black bars indicate lights off. Data are expressed as the mean + SEM (N = 6–8group). The difference with the expected new acrophase was estimated with a one-way ANOVA for repeated measures followed by a Dunnett´s post hoc test P < 0.05.

On day 6, in which rats were in DD, the control group exhibited a difference of 6.1 h between the current acrophase and the expected new acrophase (Fig. 2A), the chocolate group exhibited a difference of 2.4 h from the new acrophase (Fig. 2B), the cocoa butter had a difference of 4.8 h (Fig. 2C), gelatin of 5 h (Fig. 2D), sugar of 3.8 h (Fig. 2E) and cocoa flavor of 4.7 h (Fig. 2F). The Dunnet’s multiple comparisons test indicated a no significant difference between the current acrophase and the expected acrophase for the groups having access to chocolate and to sugar (P < 0.05).

### Chocolate for breakfast improves day-night activity in the SCN of jet-lag rats

In view of the fast entrainment observed in the CH-N rats, the effect of chocolate for breakfast on day-night pattern of c-Fos was explored in the SCN of rats exposed to experimental jet-lag (6PA).

**Figure 3:**
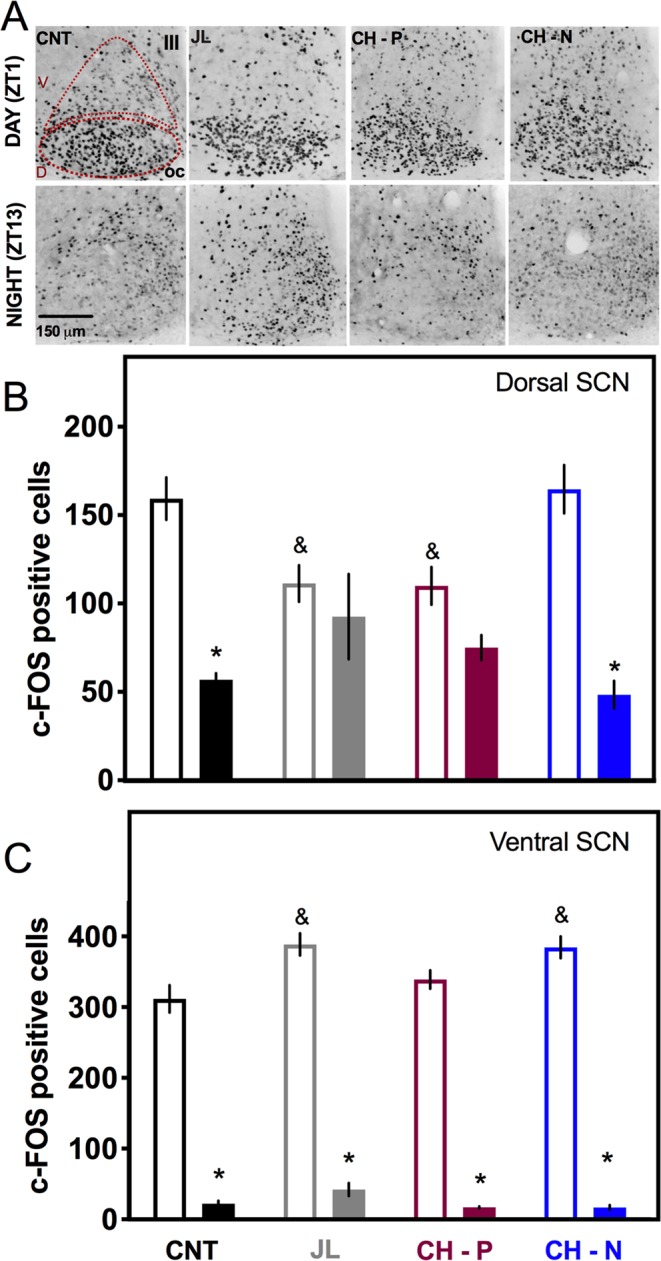
Chocolate for breakfast in jet-lag animals restored the day-night c-Fos expression in the SCN. (**A**) Representative microphotographs of c-Fos in the SCN in two time points, one hour after light onset (ZT1) and one hour after lights off (ZT13) for control rats without experimental manipulations (CNT), for jet- lag rats without chocolate (JL), jet-lag rats that received chocolate coupled to the previous night (CH-P) or jet-lag rats that received chocolate coupled with the new night (CH-N). (**B**) Number of c-Fos positive cells in the dorsomedial SCN and (**C**) in the ventrolateral SCN at ZT1 (empty bars) and at ZT13 (filled bars). Data are expressed as the mean + SEM (N = 5–6/group). The Tuckey post hoc test indicated statistical difference * day vs night, & difference from CNT (P < 0.05).

Under baseline conditions and before the 6PA, control rats (CNT) exhibited a day-night difference of c-Fos immunoreactivity in the SCN (P < 0.01), characterized by high c-Fos levels early in the light phase (Zeitgeber time 1, ZT1) and low c-Fos levels in the early night (ZT13). One day after the 6PA, this day-night c-Fos difference was lost in the dorsal region of the SCN for the JL and the CH-P groups (Fig. 3A,B). Contrasting, chocolate intake coinciding with the onset of the new night (CH-N group) induced a day-night rhythm in c-Fos expression in the dorsal SCN (P < 0.01) as observed in the CNT rats. The two-way ANOVA indicated significant interaction for time of the day x chocolate treatment (F_(3,38)_ = 7.991; P = 0.0003). In all groups, the ventral SCN responded directly to the light onset with high c-Fos levels in the early day and no differential effect was observed associated with the chocolate timing (Fig. 3C). The two-way ANOVA indicated significant interaction for time of the day x chocolate treatment (F_(3,38)_ = 3.701; P = 0.0019).

### Chocolate for breakfast prevents internal desynchronization in a shift-work model

**Figure 4:**
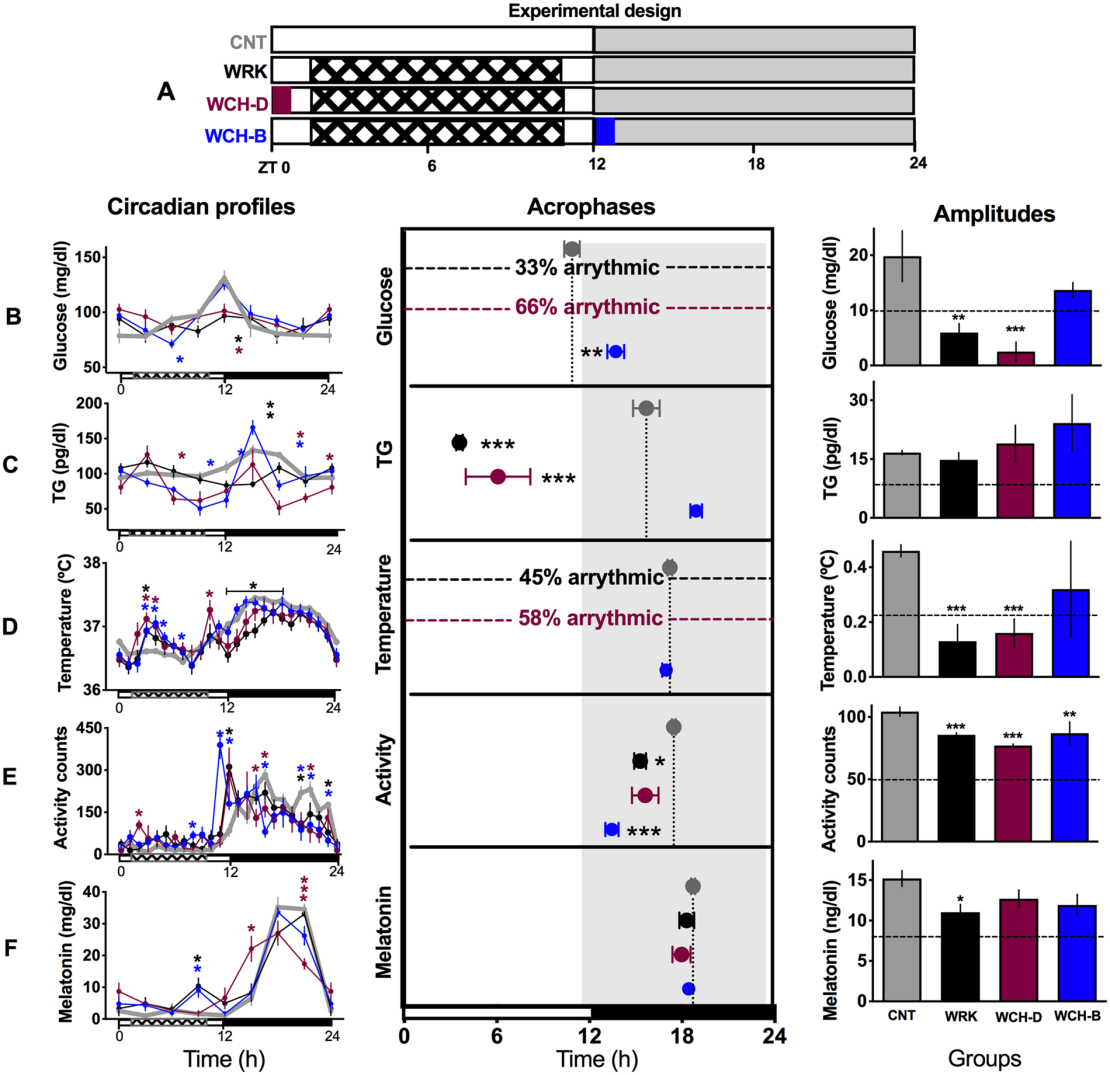
Chocolate for breakfast prevents internal desynchronization. (**A**) Experimental design for shift-work and chocolate access. The control group (CNT, grey), was not exposed to experimental manipulations, the shift-worker group exposed to 8 h forced activity without receiving chocolate (WRK, black), the shift-worker group receiving 5 g of chocolate for dinner at the onset of the rest phase (WCH-D, red) and the shift-worker group receiving chocolate for breakfast at the onset of the active phase (WCH-B, blue). Daily circadian profiles for (**B**) glucose, (**C**) triglycerides (TG), (**D**) temperature, (**E**) general activity and (**F**) melatonin were obtained during the last week of the shift-work protocol (3^rd^ week, left panels). Acrophase maps (middle panels) represent the peak level for each rhythm /group obtained with the Cosinor analysis. Dotted vertical lines indicate the acrophase for the CNT group. The amplitude (right panels) indicate the robustness of the daily rhythm. Data are represented as the mean + SEM (N = 4–6/group). For the circadian profiles (right panel), the Tuckey posT hoc test indicated statistical difference from the CNT, black asteriks WRK vs CNT, red asterisks WCH-D vs CNT and blue asterisks WCH-B vs CNT. For the acrophases and amplitudes (middle and left panels respectively), the Dunnet post hoc indicated statistical difference from CNT, black asteriks. *P < 0.05, **P < 0.001 and ***P < 0.0001.

Shift-work produces a chronic condition of internal desynchronization because individuals are forced to be active when their biological clock dictates rest^14,16^. In a shift-work model, based on forced activity during the rest phase, rats develop circadian disruption associated with metabolic dysfunction and overweight^17^. Restricting food intake to the night, prevented internal desynchronization^10^. This same shift-work model, based on forced activity in the rest-phase, was used to test whether a daily piece of chocolate for breakfast, prevented circadian disruption (Fig. 4A).

After 4 weeks of experimental shift-work (WRK), rats developed internal desynchrony characterized by the loss of rhythmicity in circulating glucose and in body temperature and a shifted daily peak of triglycerides (TG, Fig. 4B–D left column), while the daily pattern of activity and melatonin were not shifted (Fig. 4E and F left column). A daily 5 g piece of chocolate scheduled to the onset of the nocturnal activity (breakfast, WCH-B) restored the daily peak of glucose, of TG and of core temperature, while a piece of chocolate at the beginning of the rest phase (dinner, WCH-D) maintained the disrupted circadian pattern observed in the WRK group. The two-way ANOVA for RM indicated significant effects for the interaction time x chocolate treatment for glucose (F_(21,126)_ = 3.91; P < 0.0001), for temperature (F_(72,864)_ = 2.72; P < 0.0001), for TG (F_(21,126)_ = 7.63; P = 0.0.0001), for general activity (F_(72,480)_ = 4.16; P < 0.0001) and melatonin (F_(24,144)_ = 5.375; P < 0.0001).

The shift-work protocol alone or combined with chocolate scheduled for dinner lead to an arrhythmic pattern and loss of the acrophases for glucose and body temperature (Fig. 4B,D middle column). The beneficial effect of chocolate for breakfast in the shift-work group (WCH-B) was also observed in the acrophases that were restored to the night phase to similar values as the control rats (represented by the vertical dotted line, Fig. 4 middle column). The one way ANOVA indicated a significant difference among groups for glucose (F_(3,16)_ = 358.3; P < 0.0001), for TG (F_(3,16)_ = 45.21; P < 0.0001), for body temperature (F_(3,34)_ = 5261; P < 0.0001), for general activity (F_(3,20)_ = 9.976; P = 0.0003), but no effects on melatonin (F_(3,18)_ = 0.42; P = 0.73). Also, chocolate for breakfast increased the amplitude to similar values as the control group for glucose and body temperature, while activity was decreased and melatonin was not affected (Fig. 4B–F right column). Importantly, in the shift-work model chocolate for dinner (WCH-D) affected daily rhythms in a similar way as the work schedule alone (WRK).

In non-worker rats, with no risk of circadian disruption, chocolate neither for breakfast nor for dinner influenced acrophases of daily rhythms. However, CH-B enhanced the nocturnal peak of glucose and TG (Supplementary Fig. 2B,C left panel). Both chocolate schedules reduced the amplitude of general activity (Supplementary Fig. 2).

### Chocolate for breakfast improves the day-night activity in the SCN of shift-work rats

**Figure 5:**
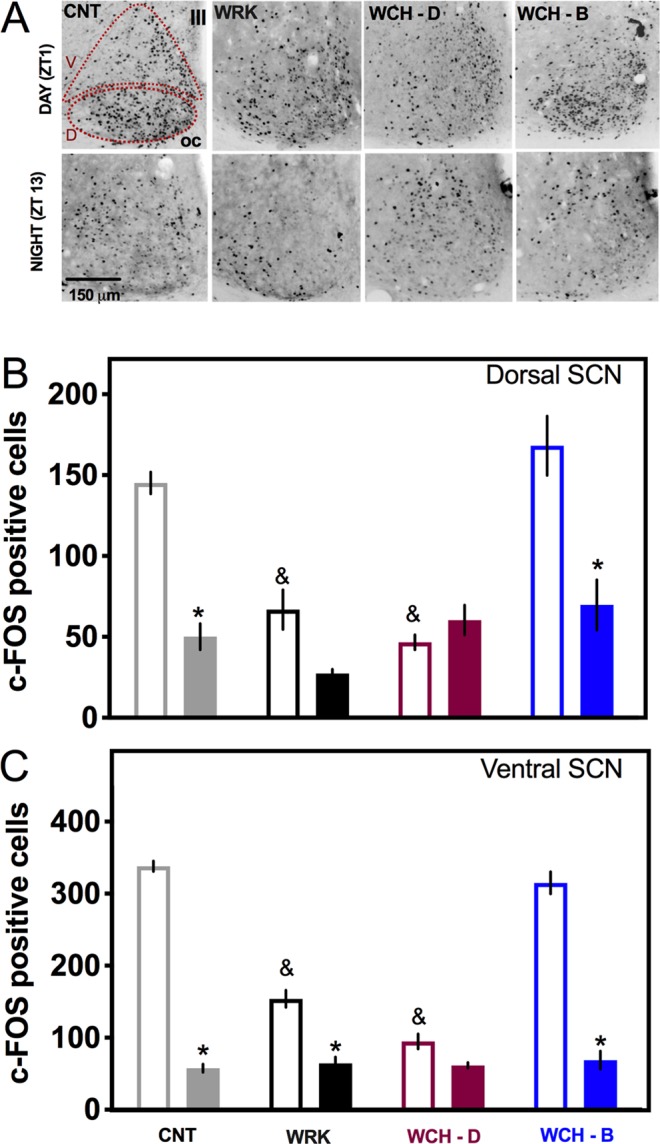
Chocolate for breakfast imposes a day-night pattern of c-Fos activation in the SCN of rats exposed to the experimental model of shift-work. (**A**) Representative SCN sections of brains obtained from control (CNT), shift-work (WRK), shift-work + chocolate for dinner (WCH-D) and shift-work + chocolate for breakfast (WCH-B) rats at two time-points, one hour after light onset (empty bars = ZT1) and one hour after lights off (filled bars = ZT13). (**B**) Number of c-Fos positive cells in the dorsomedial SCN and (**C**) in the ventrolateral SCN at ZT1 and at ZT13. Data are expressed as the mean + sem (N = 4–8/group). The Tuckey post hoc test indicated statistical difference * day vs night, and & difference from CNT, P < 0.05.

The prevention of circadian disruption observed in WCH-B rats suggested direct effects on the SCN. Therefore, the day-night pattern of c-Fos was evaluated in the SCN of rats exposed to the shift-work protocol without chocolate and with access to chocolate either for breakfast or for dinner (Fig. 5).

After 4 weeks in the forced activity protocol the brains of the WRK and WCH-D rats exhibited a significant decreased activation in the ventral and dorsal regions of the SCN during the rest phase as compared with the CNT group (Fig. 5B,C). Chocolate for dinner induced a loss of rhythm in both SCN regions of the WCH-D rats. Contrasting, chocolate for breakfast induced in the WCH-B group a clear day-night pattern in the number of c-Fos positive cells in both regions of the SCN as observed in the CTL group (Fig. 5B,C). The two-way ANOVA indicated significant interaction for time of the day X chocolate treatment for the dorsal SCN (F_(3,39)_ = 12.83; P < 0.0001) and for the ventral SCN (F_(3,39)_ = 73.74; P < 0.0001).

In non-working rats, chocolate for dinner (CH-D) also induced a loss of day-night c-Fos rhythms in the dorsal SCN with low values during the rest-phase as compared to CNT rats. Rats receiving chocolate for breakfast (CH-B) exhibited similar day-night patterns in the ventral SCN as the CNT group (Supplementary Fig. 3).

### Chocolate for breakfast or chocolate for dinner: a different outcome for body weight and differential thermogenesis

**Figure 6:**
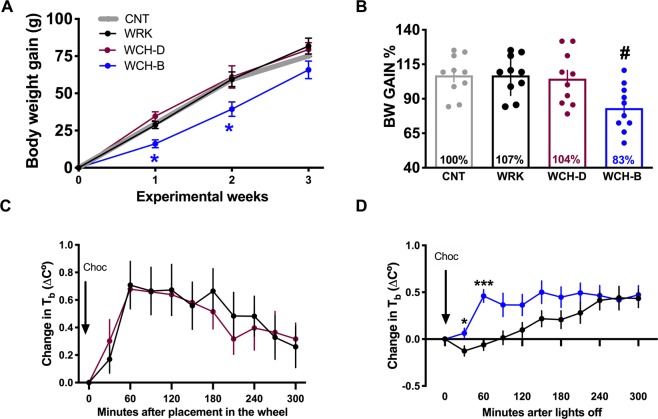
Chocolate for breakfast reduced body weight gain and promoted thermogenesis in shift-work rats. (**A**) Body weight (BW) gain for 3 weeks in control undisturbed rats (CNT, grey), in shift-worker rats exposed to 8 h of forced activity without chocolate access (WRK, black), shift-worker rats receiving 5 g of chocolate for dinner at the onset of the rest phase (WCH-D, red) and shift-worker rats receiving chocolate for breakfast at the onset of the active phase (WCH-B, blue). (**B**) Body weight gain, as compared with the CNT group (100%) at the end of the 3^th^ experimental weeks. Data are expressed as the mean + SEM (N = 10/group). For A, the Tuckey post hoc test indicated statistical difference * from CTRL. For B the Bonferroni post hoc test indicated statistical difference # from all groups (P < 0.05). Postprandial thermogenesis for the 5 h following chocolate intake (**C**) for dinner and (**D**) for breakfast as compared with the WRK group that did not receive chocolate. Data are represented every 30 minutes as the difference (**C**) from the time point prior to chocolate intake. Arrow indicates the moment when WCH-D or WCH-B received 5 g of chocolate. The Sidak post hoc test indicated difference from WRK rats, *P < 0.05, **P < 0.01.

An important consequence of a desynchronized circadian physiology is the disturbance in energy balance^2^. After 3 weeks in the protocol the WRK and WCH-D groups had attained similar body weight gain, while WCH-B rats had gained less weight as compared with all groups (Fig. 6A). The two-way ANOVA indicated significant interaction for weeks of protocol x chocolate schedule (F_(9,108)_ = 2.13; P = 0.032). At the end of the protocol WRK and WCH-D groups has reached 7% and 4% more body weight gain than the CNT group, respectively (Fig. 6B). Importantly rats that received chocolate for breakfast had gained 17% less body weight than the CNT group and 24% less than the WRK group (Fig. 6B). The one-way ANOVA indicated significant difference between groups (F_(3,36)_ = 4.961; P = 0.005).

A similar effect was observed in control, non-worker rats. In non-worker rats chocolate for dinner significantly increased body weight gain to 16% more than the CNT group, while rats eating CH for breakfast attained a low body weight (Supplementary Fig. 4A,B).

In order to determine whether the presentation of chocolate at different phases induced a differential response in energy expenditure, the thermogenic effect of chocolate during the last week of the protocol was evaluated. The body temperature in the 30 min prior chocolate presentation was compared with the body temperature for the following 5 hours after chocolate intake.

The WRK and WCH-D groups exhibited a similar posprandial body temperature response, in spite that the WCH-D group had ingested chocolate (Fig. 6C), suggesting no posprandial response to chocolate during the rest phase. The two-way ANOVA indicated no significant effect for groups X time (F_(10,180)_ = 0.46; P = 0.908). Contrasting, in the active phase chocolate ingestion resulted in an increased thermogenic response of the WCH-B group as compared with the WRK group (Fig. 6D; F_(10,180)_ = 4.073; P < 0.001). Likewise, in non-worker rats CH-B induced postprandial thermogenesis while CH-D did not (Supplementary Fig. 4C,D).

## Discussion

### The entraining effects of palatable food for circadian synchrony

The circadian system is a complex network of interacting organs and brain areas that are coordinated by the SCN^18^. Recent studies show that not only the light-dark cycle, but also other external and internal cues provide temporality to the circadian system^19,20^. Specifically, feeding schedules are powerful time signals for peripheral oscillators involved in metabolic and digestive functions like liver, kidneys, gut and adipose tissue^21^. Also, brain areas involved in energy balance and motivation for food intake are entrained by food elicited signals^14,22,23^. Importantly, food does not drive circadian rhythms for all systems and organs, because the signals provided by the light-dark cycle can be predominant for some systems and brain regions including the SCN^24,25^. In this study we observed a different speed of re-entrainment between general activity and temperature daily rhythms which confirms the differential response to chocolate elicited signals by the regulatory systems involved in activity expression and temperature balance. Other studies have also reported similar difference in the speed of re-entrainment to feeding schedules in peripheral oscillators^24,26^.

Due to this complex interaction between external time signals, the beneficial entrainment effects on circadian function are seen when food coincides with the activity phase^17,27^ while adverse effects have been reported when food is provided in the rest phase^28,29^. This study provides additional evidence of the relevant impact of the timing of food intake and specifically for palatable food intake as a strong entraing factor in order to keep circadian synchrony.

Different non-photic cues have been used to accelerate re-entrainment in experimental models of jet-lag. As early as 1987, Mrosovsky and Salmon demonstrated a fast re-entrainment to a sudden 12 h shift of the light-dark cycle by exposing hamsters for 3 h to a novel running wheel associated with the onset of the new night^30^; this was confirmed by Yamanaka *et al*. (2013) in mice^7^ and by Christian and Harrington (2002) in hamsters^31^. Moreover, exposure to the wheel in the light phase or at the end of the night phase interfered with the re-entrainment efficiency in behavior and in peripheral oscillators^32,33^. All together such findings suggest that the fast re-entrainment achieved with the daily restricted chocolate intake may be partly due to the increased arousal and excitement induce by chocolate arrival.

Food elicited signals have also shown to be powerful entraining factor for the circadian system. In recent years experimental and clinical studies have demonstrated that in order to maintain a coordinated circadian function, food intake needs to be synchronized to the light-dark cycle. And that the main effects are due to a direct synchronizing effect on peripheral clocks and brain oscillators^24,34^. The time of food intake is now suggested as a chronotherapeutic strategy that can help to ameliorate the burden resulting from circadian disruption due to shift-work and jet-lag^10,11,35^. Contrasting, feeding schedules coupled to the rest phase, trigger an internal desynchrony, resulting in the loss of homeostasis, and represent a risk for metabolic health^28,29^. Here we show that palatable food scheduled for breakfast is sufficient to maintain circadian synchrony.

Palatable food has powerful reinforcing effects on the brain and behavior. High caloric diets rich in fat, sugar and/or salt are normally highly palatable for humans as well as for experimental animals. Palatability results from taste and from the energy provided by the diet. Animals exposed to palatable diets develop non-homeostatic feeding, driven mainly by hedonic aspects of food intake and by affecting the brain reward system^36,37^. Previous studies have reported that a daily piece of chocolate can drive circadian oscillations in brain areas involved in the reward system^38,39^ and can have entraining effects on behavior and on the SCN in rats under constant darkness^15^. Moreover, palatable food based on a sugar-rich as well as fat-rich food have proven to exert a strong effect as synchronizing factors on the circadian system^40^. In this study elements contained in chocolate, like cocoa butter and flavor were also effective at advancing phase, although not with the efficiency as observed with chocolate. Contrasting sugar combined with the onset of the new night turned to be an effective element for accelerating re-entrainment. This highlights the relevance of palatability as entraining factor for the circadian system. Palatability is signaled by the ventral tegmental area to corticolimbic regions, moreover, a recent study demonstrated that stimulation of direct dopamine projections to the SCN accelerate re-entrainment after a 6 h phase shift^41^.

In normal light dark (LD) conditions, scheduled food access does not shift the phase of the SCN^25,42^. However, the daily rhythms of vasopressin in the dorsomedial SCN are modulated by feeding schedules^43^. Other studies indicate that the SCN is inhibited during food anticipation and during fasting as observed with c-Fos^19,44^ or electrophysiological recordings^44–46^, while the ventral SCN is activated after refeeding as well as by light^45^.

Moreover Gallardo *et al*. (2012) described c-Fos activation in the SCN in anticipation to a high fat diet and to cheese, providing further evidence of the influence of palatable food on the SCN^47^.

Recent findings indicate that the SCN can also respond to hedonic information via dopaminergic projections from the ventral tegmental area (VTA)^41^, which may be a pathway used by palatable food. Here we report that a palatable food scheduled for breakfast can influence the activation in the SCN, as seen with c-Fos, by setting a rhythmic pattern in the dorsomedial region, which is suggested to be the anatomical site for the biological clock. This rhythmic pattern in the dorsal SCN may lead to fast re-entrainment^35^. Importantly, the beneficial synchronizing effects of palatable food on the SCN and overt circadian rhythms was gated by a time window, where chocolate in the active phase exerted strong entraining effects, while chocolate during the rest phase did not favor re-entrainment.

### Breakfast influences the circadian system and benefits metabolism

Chocolate for breakfast exerted beneficial effects on circadian function and the correction of this desynchrony was associated with improved body weight. This is in agreement with a previous study reporting in mice exposed to chronic jet-lag, that fixed 12 h restricted food access prevented overweight and metabolic disturbance. In this study however, the fixed 12 h food access did not synchronize activity rhythms because it was mainly presented out of phase of the LD cycle^48^. In control rats eating chocolate in the rest phase had an adverse effect on body weight, while chocolate for breakfast prevented overweight. Our data agree with^49^ who reported increased body weight in mice exposed to restricted acces to chocolate during the day. Other studies have also reported that in rodents the main meal scheduled during the first half of the active phase provides beneficial effects for body weight and metabolism^9,50^. Moreover, restricted food access scheduled to the rest phase can disrupt overt circadian expression, favors obesity and leads to metabolic disorders^29^.

Finally, present data indicate that the thermogenic effect to a high caloric food (chocolate) can have a differential outcome depending on the phase of ingestion. While a piece of chocolate produced a high postprandial thermogenic response during the active phase, this was not observed in the rest phase. This is in agreement with previous reports indicating that breakfast induces a strong postprandial thermogenesis leading to energy expenditure^51–53^. Al together here we provide evidence that eating chocolate at the right phase prevents circadian desynchrony and the correction of the desynchrony may improve metabolic health.

## Conclusions

The present study explored the effects of a piece of chocolate as a synchronizing factor to prevent circadian disruption under conditions of shift-work or jet-lag. We provide evidence that a piece of chocolate, a high caloric and palatable meal, exerts beneficial effects on circadian function and body weight, when it is timed to the beginning of the active phase (breakfast). The circadian synchrony was associated with beneficial effects on metabolic rhythms and body weight, thus may be an importat factor for maintaining circadian fitness in spite of the 24/7 modern lifestyle. Further studies will need to demonstrate whether chocolate for breakfast can prevent comorbidities of internal desynchronization such as the development of tumors, depression, metabolic syndrome, and others.

## Materials and Methods

### Animals and general housing

All animals were maintained in a 12:12 h LD cycle [lights on at 07.00 h, Zeitgeber time (ZT0)], constant temperature (22 ± 1 °C) and free access to food (Rodent Laboratory Chow 5001) and water, unless otherwise stated. Experimental procedures used in this study were in strict accordance with the Mexican norms for animal handling, Norma Oficial Mexicana NOM-062-ZOO-1999, which conforms to international guidelines for animal handling, and were approved by the Ethics Committee (063/2016) in the Faculty of Medicine UNAM. Experiments conform to international guidelines on the ethical use of animals; procedures were aimed at minimizing the number of animals used and their suffering.

### Experimental designs

#### Re-entrainment with chocolate after acute jet-lag

To determine whether a piece of chocolate can aid to re-entrain in jet-lag conditions, rats were randomly assigned to one of three groups that were exposed to a sudden 6 hour phase advance (6PA): 1. The control group (n = 8), exposed to the 6PA, with *ad libitum* regular food access and NO chocolate; 2. the chocolate-previous rats (CH-P; n = 8); starting with the day of 6PA they received daily a piece (5 g) of milk chocolate (Kinder chocolate Maxi, Ferrero) coinciding with the onset of the previous night; 3. the chocolate–new rats (CH-N group n = 8), starting with the day of 6PA they received daily a piece (5 g) of milk chocolate coinciding with the start of the new night. after 10 days of baseline in an LD cycle (lights on at 1900 h, = ZT0), rats were exposed to the 6PA by turning off the light 6 h earlier, at 1300 h external time. The chocolate administration continued for the 12 days following the 6PA shift. After the 6PA, the following 12 days were monitored and the number of transitory cycles for achieving the new expected acrophase were determined for general activity and core temperature using a cosinor analysis (*see cosinor analysis*). The new expected acrophase for each group was estimated by subtracting 6 hours from the values obtained in the baseline. The daily new acrophases were statistically compared with the expected acrophase using a one-way ANOVA for repeated measures followed by a Dunnett´s post hoc test. Data that resulted statistically different from the expected acrophase were classified as transitory cycles, whereas when data were statistically similar to the expected acrophase for 3 consecutive days, the re-entrainment to the new phase was classified as reached. A one-way ANOVA was used to compare the number of days required by each group to achieve re-entrainment to the new acrophase for general activity and core temperature.

#### Re-entrainment with food after acute jet-lag

Rats exposed to a sudden 6 hour phase advance (6PA) were randomly assigned to 1 of 2 experimental groups: Food access in previous night (Food-P) or Food access in the new night (Food-N). After 10 days of baseline in an LD cycle with lights-on at 0700 h and lights-off at 1900 h, all rats were exposed to a 6 hours phase advance (6PA) as previously described. The shift was accomplished by ending the light period 6 h earlier, resulting in lights-off at 1300 h new ZT12 and lights-on at 0100 h external time (new ZT0). After the 6PA both groups were exposed daily to a restricted food access of 12 h food and 12 h fasting during 10 days. The Food-P group (*n* = 12) were fasted for 12 h and received food during 12 hours in the time of their previous night, while the Food-N group (*n* = 12) were fasted for 12 h previously to 6PA, received food during 12 hours in the new night (geographic time: 1300 h to 0100 h) for 10 days after the 6PA.

The number of transitory cycles necessary for re-entrainment was determined for general activity and temperature using a cosinor analysis as described before.

#### Re-entrainment with the chocolate components after acute jet-lag

Rats were randomly assigned to one of six groups exposed to a 6PA: the control group receiving ad libitum food and was not exposed to other feeding conditions, experimental rats after the 6PA received daily either 5 g of chocolate or pellets containing the separate elements of a chocolate bar; gelatin was used as the base to prepare all the pellets, therefore, one group received only gelatin, other group received the smell and fat of chocolate provided by cocoa butter, the calories of chocolate were mimicked with sugar and the chocolate flavor was accomplished by adding cocoa flavor with Splenda to gelatin. After 5 days in this protocol rats were left in constant darkness for 24 hours without chocolate access to determine the endogenous circadian phase. The daily phase after the 6PA was compared with the expected acrophase by sustracting 6 h form the baseline acrophase. A one-way ANOVA for repeated measure was used followed by a Dunnett´s pos hoc test to determine significant differences, as described for experiment 1. On day 6 under DD the achieved acrophase was compared with the expected acrphase and the difference was used as indicator of the re-entrainment effect of the element using a cosinor analysis as previously described.

### Chocolate effects in the experimental shift-work condition

Rats were randomly assigned to a Control (CNT) or a Shift-work (WRK) condition. These groups were further subdivided in: the control group (CNT) receiving regular chow *ad libitum* during the entire experiment, the control-chocolate for dinner group (CH-D) receiving daily 5 g of chocolate immediately after lights on (ZT0) and the control-chocolate for breakfast group (CH-B) receiving daily 5 g of chocolate immediately after lights off (ZT12). In a similar way the WRK rats were subdivided in three groups: The WRK group received chow *ad libitum* during the entire experiment, the work rats receiving chocolate for dinner (WCH-D) received daily 5 g of chocolate 10 minutes before entering to the slow rotating wheel for (ZT2); the work rats receiving chocolate for breakfast (WCH-B) received daily 5 g of chocolate at ZT 12. Rats assigned to the shift-work protocol were placed in slow rotating drums (1 revolution / 3 min) that are normally used for sleep deprivation (33 cm diameter, 633 cm wide) from Monday to Friday for 8 h (from ZT2 to ZT10) during 4 weeks, as previously reported^54^. The scheduled chocolate was only provided from Monday to Friday associated with the shift-work protocol. During weekends, all rats remained undisturbed in their home cages. Rats were weighed before starting the baseline and once / week during the first 3 week of the experimental manipulations. Body weight gain was calculated for this interval and for each group. At the end of the 3rd working week rats underwent surgery to implant a jugular cannula (n = 4–6 per group) in order to obtain blood samples for a 24 h cycle.

Rats were allowed to recover during the weekend and continued their work protocol during week 4. One series of rats was used to evaluate postprandial thermogenesis. At the end of week 4 the change in core temperature was compared between WRK rats and WRK rats receiving chocolate either for breakfast or dinner.

### Monitoring of general activity and core temperature

All rats were housed in individual cages (45 × 30 × 35 cm) placed on plates with tilt sensors, in soundproof lockers with controlled lighting conditions. General activity in the home cage was continuously monitored with the tilt sensors. Behavioral events were collected with a digitized system and automatically stored every minute in a PC for further analysis SPAD9 (Data processing system, 1.1.1 version; Omnialva SA. De CV. Mexico City, Mexico) based on MATLAB. Double plotted actograms were obtained for each animal by collecting the sum of activity for 15 min intervals. Daily activity counts were fitted to a nonlinear cosinor analysis for estimating daily individual acrophases (*see cosinor analysis below)*. For each rat, normalized activity counts for the last day of the base line, and for the following days after the 6PA were analyzed individually with the cosinor analysis, to obtain the daily acrophases. In order to evaluate core body temperature, rats underwent a brief surgery to receive intra-abdominal temperature sensors (iButton Sensor- Temperature Logger; Maxim integrated products, USA) as previously described^55^. Briefly, one week before starting baseline, rats were anaesthetized with an intramuscular dose of xylazine (Procin 8 mg/kg) and ketamine (Inoketam 40 mg/kg). Under anesthesia a small incision was performed in the abdominal cavity and the temperature sensor, previously sterilized, was introduced in the peritoneum. Abdominal muscles were sutured with absorbable catgut (000) and skin was sutured with surgical suture (Atramat, International Farmaceutica, SA. de CV. Mexico). Rats were left for 1 week to recover before starting the baseline. Temperature sensors were programmed to store samples every 30 min. Data for the baseline and for the 12 following days after the 6PA were analyzed with the cosinor test to obtain acrophases as described for general activity. The acrophase for the last day of the base line was compared with the acrophases following the 6PA a one way ANOVA for repeated mesurements followed by a Dunnett’s pos thoc test for general activity. For daily temperature profiles data in the shift-work model data are presented as average per hour. Postprandial thermogenesis in response to chocolate was evaluated at the end of week 4, in a normal work day. The postprandial response was compared between WRK rats and rats receiving chocolate (WCH-B and WCH-D). The temperature change was determined every 30 min. by comparing temperature prior chocolate presentation with the temperature of the 5 h after chocolate intake for WCH-B and WCH-D rats.

### Metabolic and hormonal rhythms

At the end of week 3 of the work protocol, animals in the shift-work protocol and their controls were anesthetized with an intramuscular dose of xylazine (Procin 8 mg/kg) and ketamine (Inoketam 40 mg/kg) and cannulated in the internal jugular vein with a polyethylene silicon tube (0.025 in. i.d. and 0.047 in. o.d.; Silastic Laboratory tubing; Dow Corning Corp., Midland, MI, USA) filled with heparin (500 U/ml) as anti-coagulant as previously reported^54^. The outer end of the cannula was fixed in the back between both shoulder blades and clotted with a small needle. Rats were allowed to recover during the weekend and on Monday the work protocol was reinitiated. At the end of the 4th working week blood samples were obtained distributed in 2 days (Thursday and Friday) to cover a 24 h cycle with 3 h intervals; 4 samples were obtained one day at ZT0, ZT6, ZT12 and ZT18 and the other 4 samples were obtained the next day (ZT3, ZT9, ZT15, AND ZT21) Blood samples (500 ul) were collected in Eppendorf tubes (1.8 ml) containing a clot-activator gel and were centrifuged at 2500 r.p.m. during 10 min, serum was stored in 100 µl aliquots at −45 °C until assay. Aliquots were processed with colorimetric methods for determination of glucose, triglycerides (TG) and melatonin.

Glucose was measured using a commercial colorimetric kit (GPSL-0507, ELI Tech Clinical Systems). TG were assessed with a commercial kit (TGML-0427, ELI Tech Clinical Systems), melatonin was determined by the ELISA method, with a commercial kit (Enzyme immunoassay for the direct, quantitative determination of melatonin; of IBL International, Germany). A cosinor analysis was used to obtain daily acrophases of the metabolic variables/ individual (*see cosinor analysis*).

### Immunohistochemical staining

One series of the groups employed for the jet-lag study was used to obtain the brains in two time points. Brains were obtained 1 day after the 6PA either at their new ZT1 (one hour after the lights onset) or new ZT13 (one hour after the light offset). ZT13 also corresponded to one hour after chocolate intake for the CH-N group. For the CH-P group, night sampling was performed at ZT19, one hour after chocolate intake.

One series of rats for the shift-work protocol was used for brain collection and immunohistochemistry. On week 4 of the shift-work protocol, rats were perfused one hour after chocolate intake (ZT1) or 12 h later (ZT13) to obtain a day-night pattern.

Rats were anaesthetized with an overdose of sodium pentobarbital (Sedalphorte 65 mg/ml, Pisa, Mexico) and were perfused transcardially with 250 ml of 0.9% saline followed by 250 ml of fixative 4% paraformaldehyde in phosphate buffer saline (PBS, 0.1 M, pH 7.2).

For the night-time points rats were anesthetized under dim red light and their eyes covered. The time between anesthesia and the start of fixation was of 5–6 min. Brains were removed and post fixed for 24 h in paraformaldehyde 4%. After post fixation they were cryoprotected in 30% sucrose for 3–4 days. Brains were frozen and cut in sections of 40 μm at −18 °C. Sections were collected in four series; one series was processed for c-Fos as previously described^36^. Free floating sections were incubated in c-Fos antibody raised in rabbit (1:2500; Santa Cruz biotechnology, Santa Cruz, CA, USA) in phosphate buffer 0.1 M, pH 7.2 with 0.9% saline 1% (PBS) 0.25% gelatin (G), and 0.3% Triton X-100 (PBSGT) for 72 h in 4 °C. This was followed by incubation in secondary antibody, goat anti-rabbit (Vector Laboratories, California USA), 1:200 in PBSGT for 2 h at room temperature, followed by incubation in avidin–biotin complex (0.9% avidin and 0.9% biotin solutions; Vector Laboratories, California, USA) in PBSGT for 2 h at room temperature. Between incubations sections were rinsed three times for 10 min in PBS. Tissues were reacted with diaminobenzidine (0.01%), nickel (0.05%) and hydrogen peroxide (35 μl,/ 100 mL 30% H2O2) to obtain a blue color. Tissues were mounted, dehydrated and cover slipped with microscopy Entellan (Merck, Darmstadt, Germany).

### SCN c-Fos quantification in Jet-Lag and Shift-work animals

One medial section of the SCN per animal was selected, images were digitalized with Leica camera ICC50 HD attached to a Leica DM500. Picture were taken with an objective 20X and analyzed with ImageJ software by determining SCN ventral and dorsal regions bilaterally of the SCN. For counting c-Fos positive cells background was subtracted (50%); threshold was determined, and particle analysis was set for particles of 1.0–2.0 circularity and 20–150 pixels. The counts of the dorsal and the ventral regions were added to determine the total amount of c-Fos in the SCN. The results were graphed as total, dorsal and ventral cFos expressing cells and were compared with a two-way ANOVA for the factors groups and time (day-night).

### Cosinor analysis

The cosinor analysis was used to obtain daily acrophases for each individual, mean and standard error of the mean (SEM) were obtained / group. The cosinor analysis was performed with MATLAB version 5.3 using the least square method to fit a sine wave to a time series (in this case to 24 h). The formula used was: Y(t)=M + Acophase (2πt/τ + φ) + e(t); Y = collected data; M = mesor; A = amplitude; φ = acrophase; T = period; e = error at each time. The obtained acrophase/day was compared with the expected acrophase using the Student “t” test (significant values set at *p* < 0.05). Days when the acrophase was statistically different (indicated with asterisks) from the new expected acrophase were considered transitory cycles, while re-entrainment was achieved when no statistical difference between the mean daily acrophase and the expected new acrophase was indicated.

### Statistical analysis

Graphics and statistical analysis were elaborated using the GraphPad Prism version 6.00 for macOS, GraphPad Software, La Jolla California USA, www.graphpad.com.

## Supplementary information

Supplementary information.

## Data Availability

All data generated and analyzed as a part of this study are included within this article (and its supplementary information files).
