# Supplementary material for: Chocolate for breakfast prevents circadian desynchrony in experimental models of jet-lag and shift-work
Source: Sci Rep. 2020 Apr 10;10:6243. doi: 10.1038/s41598-020-63227-w (PMC7148329; doi:10.1038/s41598-020-63227-w)
Supplement: Supplementary file 1 — Supplementary information. [file 41598_2020_63227_MOESM1_ESM.docx]

Chocolate for breakfast prevents circadian desynchrony in experimental models of jet-lag and shift-work

Carolina Escobar^1B*,^ Estefania Espitia-Bautista^1A^, Mara A Guzmán-Ruiz^2A^, Natalí N Guerrero-Vargas^1B^, Miguel Ángel Hernández-Navarrete^1A^, Manuel Ángeles-Castellanos^1B^, Brenda Morales-Pérez^1^, Ruud M Buijs^3^

^1^ Departamento de Anatomía, Facultad de Medicina, UNAM.

^2^ Departamento de Fisiología, Facultad de Medicina, UNAM.

^3^Instituto de Investigaciones Biomédicas, UNAM.

.

^A^ These authors contributed equally

^B^ These authors jointly supervised this work

***Corresponding Author:**

Carolina Escobar

Departamento de Anatomía

Facultad de Medicina UNAM

Av Universidad 3000

Ciudad Universitaria

CDMX 04510

Fax number: 5623 2422

Telephone number: 5623 0222 ext 45062

e-mail address: escocarolina@gmail.com

**FIGURE S1**

**Supplementary Figure 1. Restricted food access during the previous night or during the new night differentially affects entrainment in a *jet-lag* protocol.** A) Experimental design for the jet-lag model and chocolate schedule. Rats were randomly assigned to one of 2 groups, all of them were exposed to a sudden 6 h phase advance (6PA) by switching off the lights 6 hours before expected, activity and core temperature were recorded. FOOD-P group received food access after the 6PA (indicated by the red rectangle) at the onset of the previous night. FOOD-N group received food access at the time of the 6PA (indicated by the blue rectangle). B) Representative actogram, C) acrophases for general activity and D) acrophases for core temperature of the FOOD-P group. E) Representative actogram, F) acrophases for general activity and G) acrophases for body temperature of the FOOD-N group. Acrophases were calculated from the baseline and for each day after the 6PA. The expected new acrophase was obtained by subtracting 6h from the baseline acrophase (dotted vertical line). Asterisks indicate significant differences from the new expected phase (Dunnett’s post hoc test; P<0.05). Horizontal white and black bars over the X-axis and over the actograms indicate the 12 h day and 12h night cycle.

**FIGURE S2**

Supplementary Figure 2. Effect of chocolate in control groups. A) Experimental design for the chocolate protocol. The control (CNT, black) group was left at their home cages for the entire protocol without disturbances, the two other experimental groups received a daily piece of chocolate (5g) at the beginning of the resting phase for dinner (CH-D red) or at the beginning of the active phase for breakfast (CH-B blue). Daily circadian profiles (left panels), for B) Glucose, C) triglycerides (TG) D) temperature, E) general activity and F) melatonin acrophases (middle panels). The dotted vertical lines indicate the calculated acrophase for the CNT group, only the animals that presented at least 50% of the amplitude of the CNT were considered rhythmic. The amplitude (right panels) indicates the robustness of the daily rhythm. Data are represented as the mean + SEM (N=4-10/group). For B-F right panel the Dunnett’s post hoc test indicated differences between CNT and CH-D (red asterisk), blue asterisks indicate difference between CNT and CH-B. For amplitudes (right panels) the Dunnett’s post hoc test indicated difference from CNT group, * P<0.05, ** P<0.001 and *** P<0.0001.

FIGURE S3


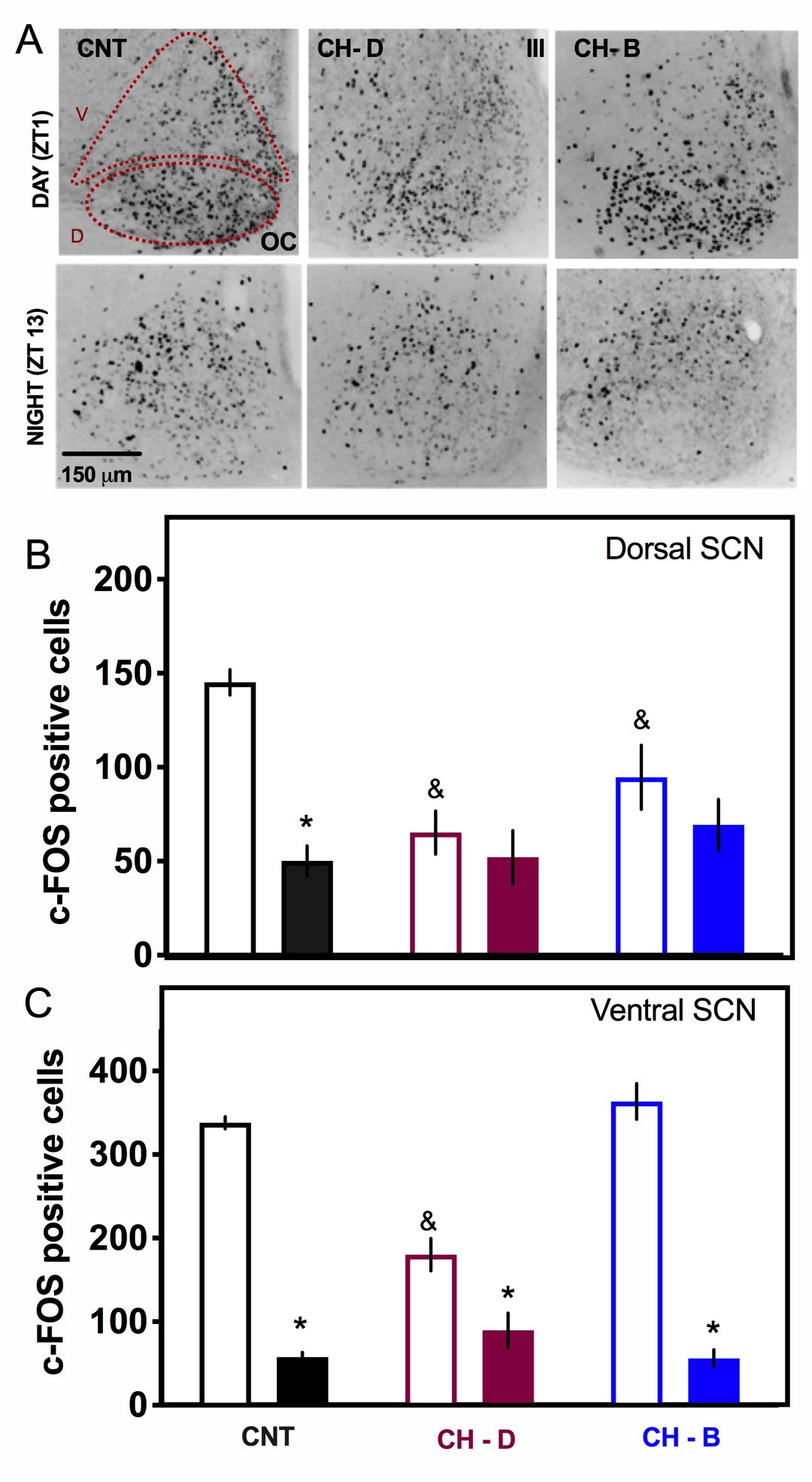


**Supplementary Figure 3. Effects of chocolate for breakfast and dinner in the SCN of control rats.** To determine whether the time of chocolate delivery modifies the day-night rhythm in the SCN we characterized c-FOS expression during the day (white bars; ZT1) and during the night (filled bars; ZT13). This analysis was performed after 4 weeks of chocolate consumption. The control (CNT, black) group was left at their home cages for the entire protocol without disturbances, the two other experimental groups received a daily piece of chocolate (5g) at the beginning of the resting phase for dinner (CH-D red) or at the beginning of the active phase for breakfast (CH-B blue). A) Representative SCN sections for each experimental group. B) dorsal SCN c-Fos expression, C) ventral SCN c-Fos expression. Data are expressed as the mean + SEM (N=5-8/group). The Tuckey post hoc test indicated statistical difference * day vs night, & difference from CNT (P<0.05).

FIGURE S4


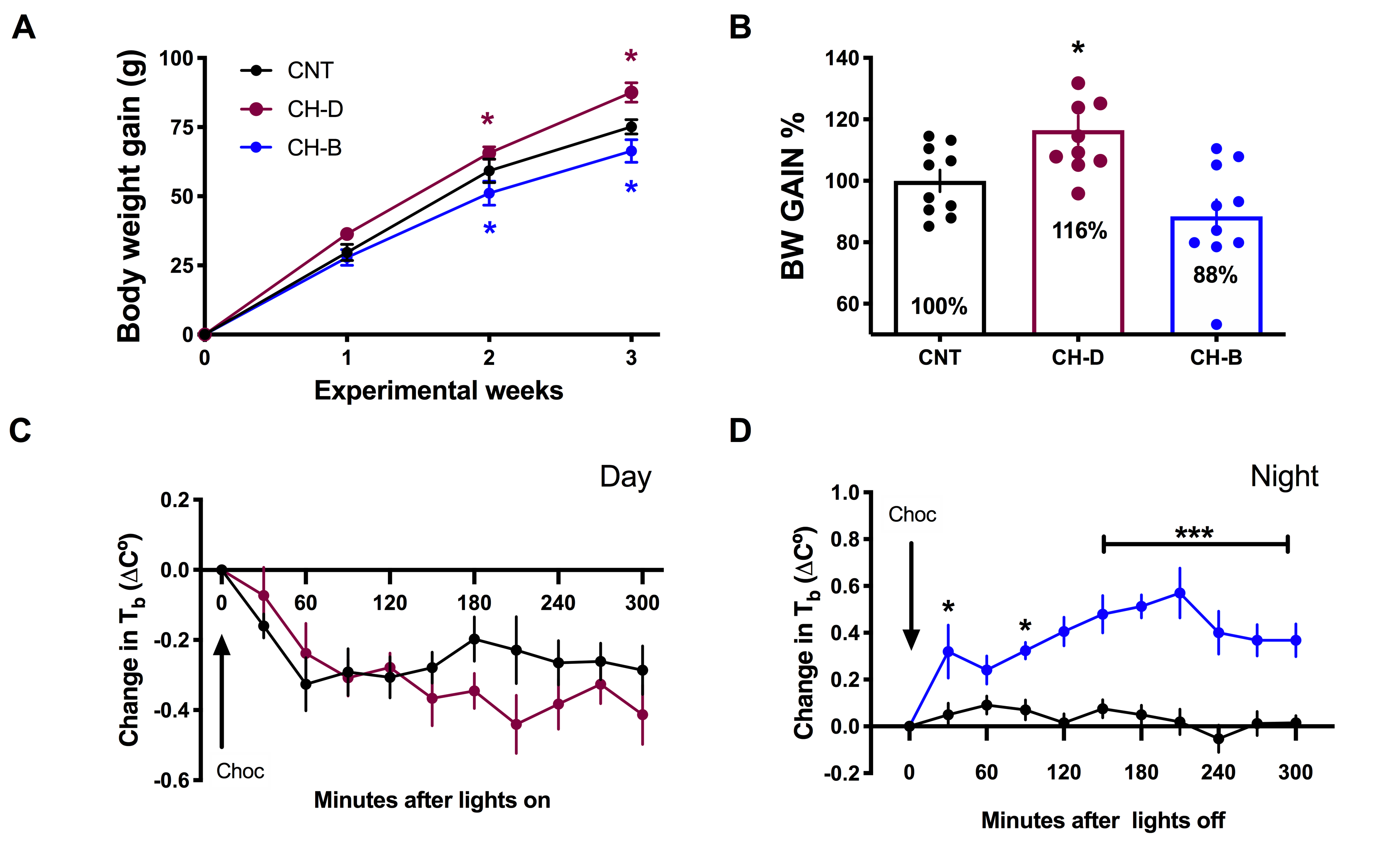


Supplementary Figure 4. Chocolate for breakfast reduce**d** **body weight gain** and promote**d** thermogenesis. Control **rats** without **access to** chocolate (CNT, black), receiv**ing** a daily piece of chocolate (5g) at the beginning of the resting phase (**dinner,** CH-D red) or at the beginning of the active phase (**breakfast,** CH-B blue). A) Body weight gain **during 3 weeks,** B) **% of** body weight gain as compared with the CNT group (100%). **C) Core temperature change in CTRL rats vs CH-D after ZT0, D) Core temperature change in CTRL rats vs CH-B after ZT12.** Arrow indicates the moment when CH-D or CH-Bgroups received 5g of chocolate.Data are expressed as the mean + SEM (N=10-11/group). For A, the Tuckey post hoc test indicated statistical difference, blue asterisk CH-B vs CNT, red asterisk CH-D vs CNT. For B the Bonferroni post hoc test indicated statistical difference from CNT group (red asterisk). For D the Sidak post hoc test indicated statistical difference form CNT. * P<0.05, and *** P<0.0001.
